# Multiple bacterial partners in symbiosis with the nudibranch mollusk *Rostanga alisae*

**DOI:** 10.1038/s41598-021-03973-7

**Published:** 2022-01-07

**Authors:** Natalia V. Zhukova, Marina G. Eliseikina, Evgeniy S. Balakirev, Francisco J. Ayala

**Affiliations:** 1 National Scientific Center of Marine Biology, Russian Academy of Sciences, Vladivostok, 690041 Russian Federation; 2 Cátedra Francisco José Ayala of Science, Technology, and Religion, University of Comillas, Madrid, Spain

**Keywords:** Fatty acids, Biodiversity, Symbiosis

## Abstract

The discovery of symbiotic associations extends our understanding of the biological diversity in the aquatic environment and their impact on the host’s ecology. Of particular interest are nudibranchs that unprotected by a shell and feed mainly on sponges. The symbiotic association of the nudibranch *Rostanga alisae* with bacteria was supported by ample evidence, including an analysis of cloned bacterial 16S rRNA genes and a fluorescent in situ hybridization analysis, and microscopic observations. A total of 74 clones belonging to the phyla α-, β-, γ-Proteobacteria, Actinobacteria, and Cyanobacteria were identified. FISH confirmed that bacteriocytes were packed with *Bradyrhizobium*, *Maritalea*, *Labrenzia*, *Bulkholderia*, *Achromobacter*, and *Stenotrophomonas* mainly in the foot and notum epidermis, and also an abundance of *Synechococcus* cyanobacteria in the intestinal epithelium. An ultrastructural analysis showed several bacterial morphotypes of bacteria in epidermal cells, intestine epithelium, and in mucus layer covering the mollusk body. The high proportion of typical bacterial fatty acids in *R. alisae* indicated that symbiotic bacteria make a substantial contribution to its nutrition. Thus, the nudibranch harbors a high diversity of specific endo- and extracellular bacteria, which previously unknown as symbionts of marine invertebrates that provide the mollusk with essential nutrients. They can provide chemical defense against predators.

## Introduction

Symbiotic associations between marine invertebrates and microbes are ubiquitous and play an important role in the ecology and evolution of species^1–3^. In the marine environment, the symbiosis of invertebrates with photoautotrophic microalgae in the photic zone and the symbiosis with chemoautotrophic bacteria, found in extreme habitats such as deep-sea water and hydrothermal vents or cold seeps, are the most widely distributed and well-studied associations^1,4^. Associations with heterotrophic bacteria are also known, although they are described to a much lesser extent. These are bone-eating polychaete of the genus *Osedax* with bacteria from the order Oceanespirillales that are known for heterotrophic aerobic degradation of complex organic compounds^5^, wood-boring mollusks with cellulose-degrading bacteria^6^, and marine polychaete with N_2_-fixing *Mesorhizobium* sp. from an anoxic microbial mat community^7^.

The discovery of new symbiosis extends our understanding of the symbiotic diversity and their impact on the ecology and evolution of the marine invertebrates. Symbiotic microbes typically play a crucial role in supplying nutrients to their hosts^8^. In addition, some of symbionts provide the hosts with chemical defense against predators and environment^2,9,10^. Symbiosis is evidently expected in inhospitable environments for invertebrates with high concentrations of methane or hydrogen sulfide^11,12^, whereas marine organisms from shallow waters have rarely been investigated for symbiotic relationships^13–15^. Meanwhile, nudibranchs, which are not protected by a shell and feed mainly on sponges, may be of particular interest.

Nudibranchs are a common element of trophic webs in the benthic marine ecosystems. Found in almost all marine habitats, from the intertidal zone to the deep-sea, they have undergone a great evolutionary radiation, clearly influenced by dietary adaptation. A number of nudibranch species, belonging to two groups, Aeolidoidea and Dendronotoidea, have symbiotic relationships with photosynthetic dinoflagellates. They usually acquire them by sequestration from their prey and keep them inside cells of digestive gland^16–18^. For the first time, the presence of symbiotic bacteria in the mucus surrounding the egg capsules as well as between and partly aligned with the microvilli, in the vestibular gland of *Dendrodoris nigra* was detected by histological and ultrastructural analysis^19^.

The high abundance of odd and branched fatty acids specific to bacteria in their tissues led us to the hypothesis that nudibranchs may house symbiotic bacteria^20,21^. The nudibranch *Dendrodoris nigra* was found to harbor symbiotic bacteria enclosed in secondary vacuoles in the epithelial cells of the notum and the mantle edge^20^. Although these data support the hypothesis, they also raise many questions as to the prevalence of bacterial associations among nudibranch species, identification of specificity of bacteria, and determination of their responsibility in mollusks.

**Figure 1:**
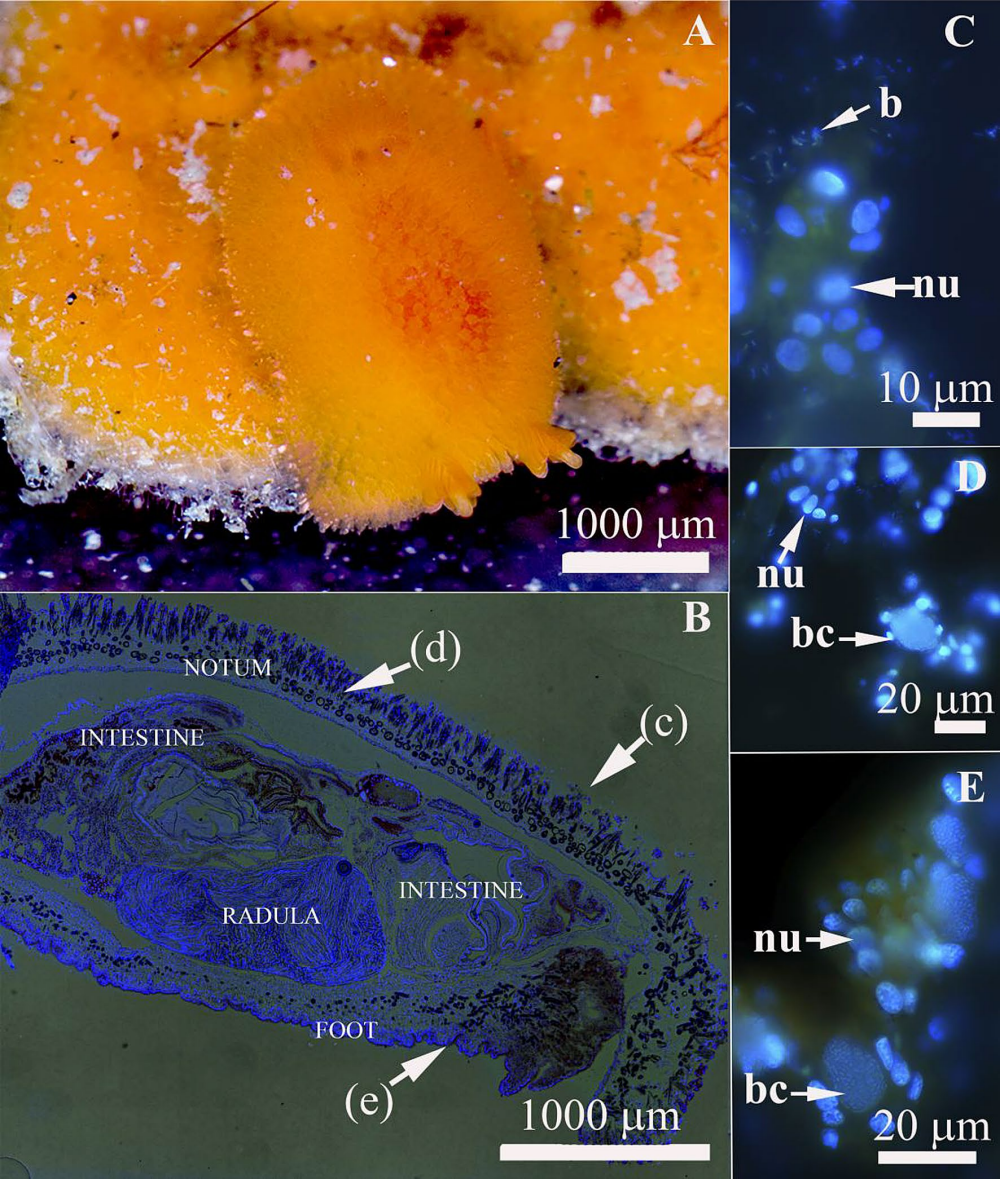
Morphology and localization of symbionts in *Rostanga alisae*. (**A**) Common view of the nudibranch in the natural habitat on the sponge. (**B**) Histological organization, longitudinal section. (**C–E**) are enlarged views of the areas indicated by the corresponding arrows: **(C)** Bacteria (b) in the mucus layer covering the body. Bacterial clusters (bc) in the epidermis of the notum (**D**) and foot (**E**). Nuclei (nu) of epithelial cells are visible. Combination of fluorescence microscopy and Phase microscopy, DNA stained with DAPI (blue fluorescence).

The present study focuses on the nudibranch mollusk *Rostanga alisae* Martynov, 2003, a member of the family Discodorididae that inhabits the intertidal zone to a depth of 15 m in the northwestern Sea of Japan and preys exclusively on the red sponge *Ophlitaspongia pennata* (Lambe, 1895) from the family Microcionidae (Fig. 1A).

The hypothesis that *R. alisae* harbors symbiotic bacteria was tested by molecular phylogenetic analyses, epifluorescence microscopy, transmission electron microscopy, fluorescent in situ hybridization (FISH) analysis, and fatty acid analyses of tissues. This investigation was aimed to assess the diversity and phylogenetic composition of symbionts’ populations and to assign the nature of the nudibranch–bacteria symbiosis. The results provide evidence for an endo- and extracellular bacterial consortium, which is more complex than other marine symbioses described before. The results are discussed with a special emphasis on the bacterial diversity and on the potential role of the discovered symbionts. This study is the first multifaceted investigation of symbiotic association of bacteria with a nudibranch species.

## Methods

### Specimens

Specimens of the nudibranch *R. alisae* (Fig. 1A) and sponge *O. pennata* were collected in the Peter the Great Bay, Sea of Japan, by SCUBA diving, at the depth of 1–6 m year-round in 2017–2019. They were then kept in a tank or used immediately for analyses. The nudibranchs were dissected by razor and separate tissues were excised for the analyses under stereomicroscope Leica ES2 (Germany).

### DNA amplification, cloning, and sequencing

Total genomic DNA was extracted from the foot, intestine, and notum tissues using the DNeasy Plant MiniKit protocol (Qiagen, Hilden, Germany). The procedures of DNA amplification, cloning, and sequencing were described previously^13,14^. A 1.5-kb fragment of the 16S rRNA bacterial genes was amplified with universal primers^22^: 5’-agagtttgatcatggctcag-3’ (27F, forward) and 5’-ggttaccttgttacgactt-3’ (1492R, reverse). The PCR reactions were carried out in final volumes of 25 µl using TaKaRa Ex Taq™ in accordance with the manufacturer’s description (Takara Biotechnology Co., Ltd.). The PCR reaction mixtures were placed in a DNA thermal cycler (Eppendorf, Mastercycler Gradient), incubated for 5 min at 94 °C and subjected to 32 cycles of denaturation, annealing, and extension: at 94 °C for 30 s, at 52 °C for 30 s, and at 72 °C for 1.5 min, with a final 7-min extension period at 72 °C. The PCR products for the 16S rRNA gene were cloned (TOPO TA cloning kit, Invitrogen, Calif.) and sequenced by the dideoxy chain-termination technique using a Dye Terminator chemistry and separated on an ABI PRISM 377 automated DNA sequencer (Perkin Elmer). The sequences of both strands were determined for each clone, using overlapping internal primers spaced, on average, 500 nucleotides. At least two independent PCR amplifications were sequenced in both directions to correct for possible cloning or sequencing errors. The 16S rRNA sequences have been deposited in GenBank under accession numbers MZ410589–MZ410616. The sequences were assembled using the program SeqMan (Lasergene, DNASTAR, Inc.). Multiple alignment was carried out manually and using the program CLUSTAL W^23^. Putative chimeras were identified using the Bellerophon software^24^. Two programs available online, RDP Classifier^25^ and Greengenes^26^, were used to uncover the bacterial affinities. The maximum-likelihood tree of the 16S rRNA sequences of *Rostanga alisae* symbionts was constructed with the program IQ-TREE^27^ using the TIM3 + F + I + G4 model of nucleotide substitution.

### Transmission electron microscopy (TEM)

For TEM, the tissue samples were fixed with a 2.5% glutaraldehyde solution in a 0.1 M cacodylate buffer (pH 7.2) for 4 h at room temperature. After being rinsed in the buffer, the samples were post-fixed for 1 h with a 1% osmium tetroxide solution and dehydrated in a graduated series of ethanol and acetone, and then embedded in a mixture of Epon and Araldit epoxy resin (Sigma, USA). Ultra-thin sections (ca. 50 nm) were prepared on a Leica EM UC6 ultramicrotome, post-stained with a 0.5% uranyl acetate and lead citrate solution, and viewed using a Libra 120 electron microscope (Carl Zeiss, Germany). For TEM, 15 nudibranch individuals were used.

### Fluorescent in situ hybridization (FISH)

For the FISH analysis, the foot, intestine, and mantle tissues were initially fixed at 4 °C in a 4% paraformaldehdye solution (pH 7.0) in a phosphate-buffered saline (PBS) for 8 h. The fixed samples were rinsed 3 × 1 h, impregnated with a 15% sucrose solution on PBS, placed in NEG 50 ™ (Thermo Scientific, USA), and frozen at –50 °C. The frozen tissues were sectioned (12–14 µm thick) on a Histostar 500 HM (Thermo Scientific) and placed onto glass slides treated with polylysine. A bacterial universal probe EUB338 was used along with a group of specific probes designed as symbiotic bacteria dominant in tissues of *R. alisae* (Table S1). Hybridization was performed at 46 °C for 3.5 h in a solution containing 900 mM NaCl, 20 mM Tris/HCl, 35% formamide, 0.01% SDS, and 3 ng/ml of the EUB338 probe along with one of the specific probes. After hybridization, the slides were washed twice at 48 °C for 15 min in a buffer containing 100 mM NaCl, 20 mM Tris/HCl (pH 8.0), 5 mM EDTA, and 0.0001% SDS. The sections were stained with a dilute 4′6'-diamidino-2-phenylindole (DAPI) solution (5 µg ml^–1^) for 1 min and examined by epifluorescence microscope using a LSM 780 laser scanning confocal microscope. For FISH microscopy, 5 nudibranch individuals were used.

### Lipid analysis

Lipid extracts were prepared from homogenized tissues according to^28^. Fatty acid methyl esters (FAME) were prepared by the treatment of lipids with 2% H_2_SO_4_/MeOH in a screw-capped vial (2 h, 80 °C) under argon and purified by preparative TLC on silica gel in benzene. FAMEs were analyzed on a Shimadzu GC-2010 chromatograph equipped with a flame ionization detector and a capillary column (Supelcowax 10, 30 m × 0.25 mm i.d.) at 210 °C. The injector and detector temperatures were 250 °C. Fatty acids were identified by comparison with equivalent chain length values and confirmed by GC–MS of their FAMEs and DMOX derivatives on a GCMS QP5050A Ultra instrument (Shimadzu, Kyoto, Japan) (electron impact at 70 eV) fitted with a MDN 5S capillary column (30 m × 0.25 mm). The carrier gas was He at 30 cm s^−1^. The GC–MS analysis of FAMEs was performed at 160 °C increased to 260 °C with a 2 °C min^−1^ ramp. The GC–MS analysis of DMOX was performed at 210 °C with a 3 °C min^−1^ ramp to 270 °C held for 40 min. For fatty acid analysis, 7 nudibranch individuals were used.

### Statistical analysis

Significance of differences in mean contents of fatty acids between the nudibranch tissues and the sponge was tested by one-way analysis of variance (ANOVA). Statistical analysis was performed using STATISTICA 5.1 (StatSoft, Inc., USA). A statistical probability of *p* < 0.05 was considered significant. Values are represented as mean ± standard deviation, n = 7.

## Results

### Histological analysis

Morphological analysis using a differential interference contrast (DIC) and epifluorescence microscopy of tissues (Fig. 1B–E) revealed bacterial clusters (10 − 15 µm overage size), and individual single bacteria in the epidermis covering the notum (Fig. 1D) and foot (Fig. 1E). Bacteria were also visualized in the mucus layer covering the body surface (Fig. 1C).

### Genetic affiliation of the bacterial 16S rRNA clones

**Figure 2:**
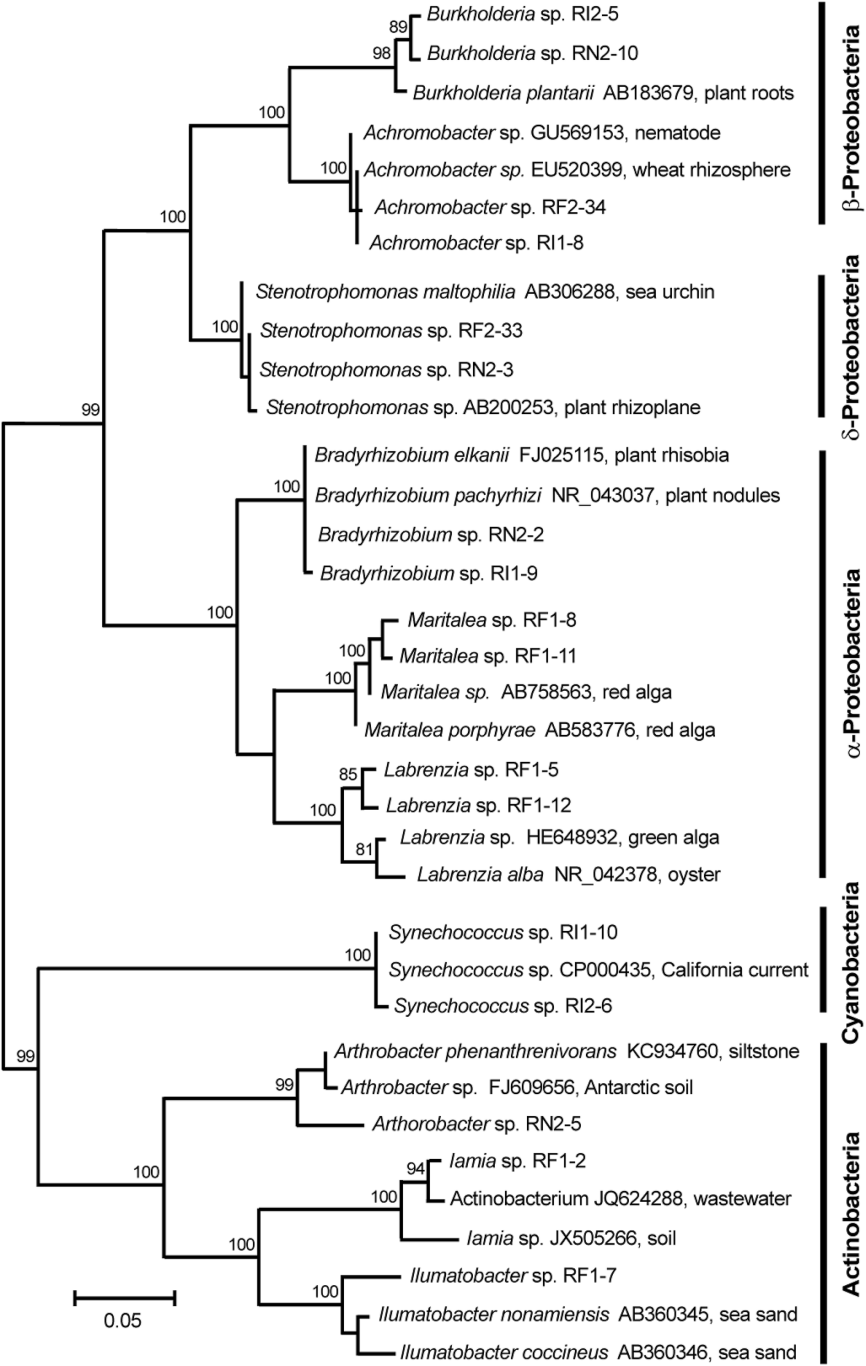
Maximum-likelihood tree of the 16S rRNA sequences of *Rostanga alisae* symbionts. The tree is based on the TIM3 + F + I + G4 model of nucleotide substitution. The numbers at the nodes are bootstrap percent probability values based on 10,000 replications (values below 75% are omitted). The number following the name of specimens (RF, RN, and RI) denotes the clone number. *RF* foot, *RN* notum, *RI* intestine.

The analysis of the cloned bacterial 16S rRNA genes allowed to obtain the most conservative and reliable scan of bacterial diversity, which made it possible to reveal bacteria that form mutualistic relationships with the host and to formulate the most parsimonious hypothesis concerning the functional importance of the symbiosis. On the basis of non-chimeric sequences (a total of 74 clones), 24 bacterial phylotypes were detected (with 97% identity cutoff) in tissues of *R. alisae* (Table S2). Phylogenetic analysis of each clone showed close matches with a multitude of bacteria (Fig. 2) belonging to the classes α-Proteobacteria (15 clones), β-Proteobacteria (13 clones), γ-Proteobacteria (16 clones), Cyanobacteria (19 clones), and Actinobacteria (7 clones). Three rare phylotypes were associated with the classes δ-Proteobacteria (1 clone), Planctomycetes (1 clone), and Fusobacteria (2 clones). Three phylotypes (Bradyrhizobiaceae, Rhodobacteraceae, and Alcaligenaceae) were classified at the family level only. The rest of the phylotypes (except for those represented by single clones) belonged to 13 genera: *Bradyrhizobium*, *Maritalea*, *Labrenzia*, *Burkholderia*, *Achromobacter*, *Stenotrophomonas*, *Synechococcus*, *Arthrobacter*, *Ilumatobacter*, *Aquicella*, *Lysobacter*, *Legionella*, and *Leptotrichia* (Table S2).

The phylogenetic analysis placed the bacterial phylotypes recovered from *R. alisae* within a clade of α-Proteobacteria that contains *Labrenzia*, *Maritalea*, and *Bradyrhizobium*. Phylotypes of symbiotic *Labrenzia* (transferred from *Stappia*) belong to a single cluster with the other species and strains of this genus. The closest relative of the nudibranch’s symbiont *Labrenzia* is *L. alba* isolated from oyster and described as straight rods, motile by a subpolar flagellum, and chemoorganotrophic aerobe^29^. One of the three α-Proteobacterial symbionts was clustered with *Maritalea porphyrae* (transferred from *Zhangella*), isolated from the red alga *Porphyra yezoensis*^30^. Another member of α-Proteobacteria symbionts was clustered with *B. pachyrhizi* and *B. elkanii*, which were isolated from nodules of legume plants. Among the dominant β-Proteobacterial symbionts, one fell into the genus *Burkholderia* and another into *Achromobacter*. The dominant γ-Proteobacteria symbiont was phylogenetically located within the genus *Stenotrophomonas*, whose species are found ubiquitously worldwide, particularly in close association with plants. The cyanobacterial 16S rRNA sequences from the nudibranch were grouped with a clade of sequences of marine *Synechococcus* spp. isolated from the water column. A sequencing analysis and BLAST searches retrieved close relation between nudibranch symbionts and actinobacteria *Arthrobacter*, *Iamia*, *Ilumatobacter*, and *Kocuria*.

Mosaic sets of common and tissue-specific bacterial phylotypes were detected in the foot, notum, and intestine. The clones were unequally distributed between the tissues studied. The deviation from equal proportions was highly significant (χ^2^ = 25.33, df = 1, *P* < 0.001). *Synechococcus* (Cyanobacteria) had the most abundant clones, especially in the intestine. *Maritalea*, *Labrenzia*, and a member of Rhodobacteriacea were located exclusively in the foot, which was especially rich in bacterial clusters. They, consequently, were specific symbionts of the foot tissue. *Bradyrhizobium* and *Burkholderia* were predominantly detected in the notum. Phylotypes of *Stenotrophomonas* were localized exclusively in the integumentary tissues, foot, and notum, and only *Achromobacter* clones were evenly distributed over the tissues. Phylotypes of Actinobacteria were found only in integumentary tissues; therefore, the species-specific tissue distribution was evident. Thus, *Iamia*, *Ilumabacter*, and *Kocuria* were found only in the foot; in contrast, another member of Actinobacteria, *Arthrobacter*, could be considered as a specific bacterium of the notum. Other bacterial clones had mosaic distribution with no clear preference for a tissue.

In addition to predominant bacterial phylotypes, members of γ-Proteobacteria such as *Coxiella*, *Aquicella*, *Acinetobacter*, and *Aliivibrio*, as well as *Desulfovibrio*, *Planctomycetes*, and *Leptotrichia*, were also present as single clones mainly in the intestine. These are known as intracellular parasites of protozoa and animal cells^31^, which suggest that these species are unlikely to be true symbiotic bacteria, and their presence most probably resulted from the contamination of this tissue as reported for *Aquicella siphonis* parasitizing an amoeba^31^.

### FISH observations

**Figure 3:**
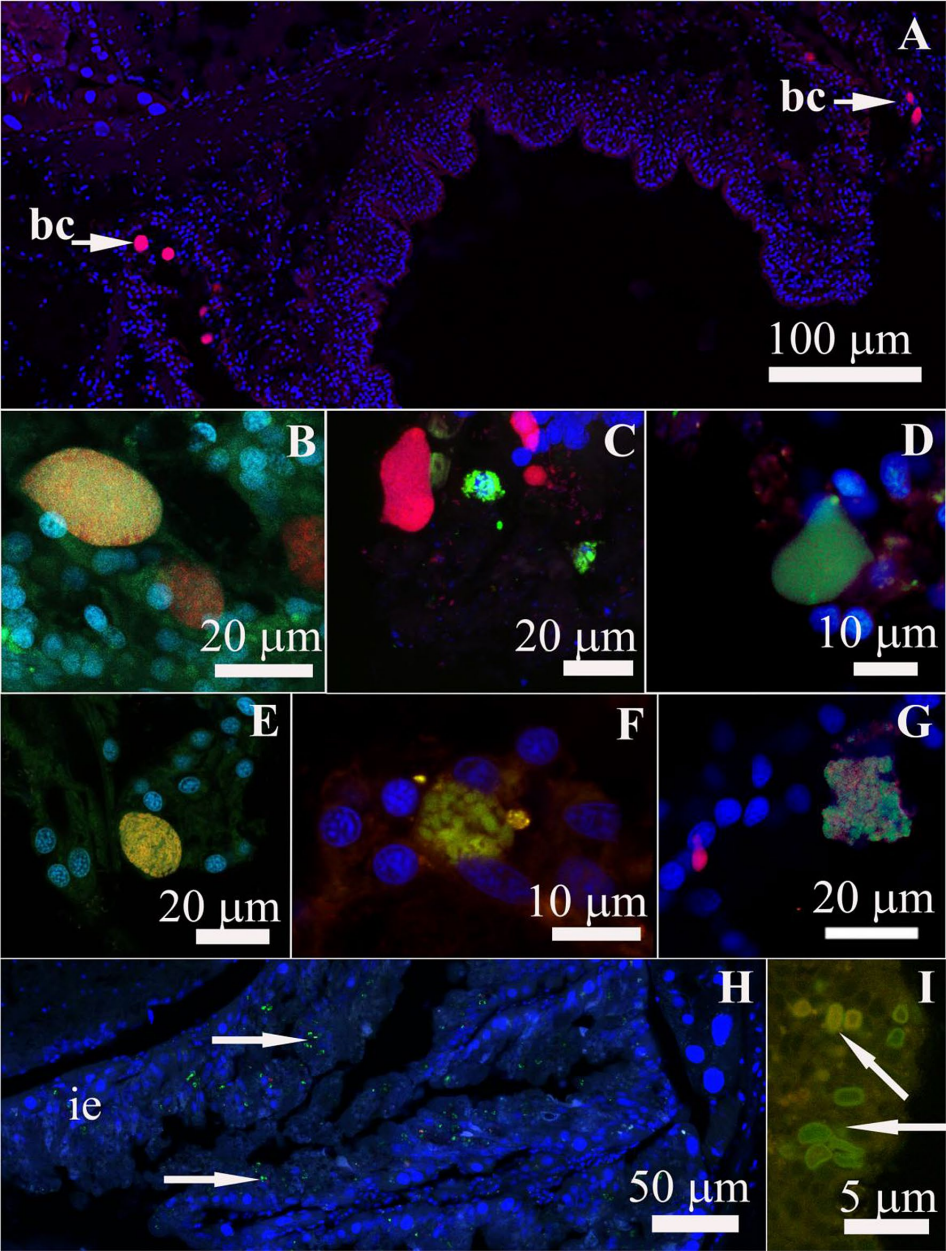
Fluorescent in situ hybridization microscopy of bacteria forming bacteriocytes (bc) in the epidermis and cyanobacteria in the intestinal epithelium of *Rostanga alisae*. (**A**) Accumulation of bacteriocytes (bc) in the skin fold between the foot and notum, transverse section of the body. Red fluorescence of bacteria hybridized with Cy3 universal UEB388 probe set. Green fluorescence of bacteria hybridized with bacteria-specific probes for (**B**) *Achromobacter*, (**C**) *Stenotrophomonas*, (**D**) *Labrenzia*, (**E**) *Maritalea*, (**F**) *Bradyrhizobium*, (**G**) *Burkholderia*, **(H,I)** *Synechococcus* (arrows). DNA stained with DAPI (blue fluorescence).

A bacterial universal probe EUB338 revealed that the bacterial clusters were more abundant in cytoplasm of the foot epithelium in the skin fold between the foot and the notum (Fig. 3A). The presence and localization of bacterial symbionts in tissues was confirmed by FISH analysis using the genus-specific oligonucleotide probes (Table S1) for *Maritalea*, *Labrenzia*, *Bradyrhizobium, Burkholderia*, *Achromobacter*, *Stenotrophomonas*, and Cyanobacteria (Fig. 3B–I).

The group-specific probes for *Achromobacter* and *Stenotrophomonas* showed discrete patches of hybridization in epithelial cells of the foot and notum (Fig. 3B,C), whereas oligonucletide probes for *Labrenzia* and *Maritalea* hybridized strongly with bacteria that settle in the foot epithelium (Fig. 3D,E). Bacterial signals of *Bradyrhizobium* and *Burkholderia* were found only in notum epithelial cells (Fig. 3F,G). Each of the symbionts was localized in clusters and occurred within a single cluster. FISH analysis also revealed the presence of cyanobacteria in the epithelial cells of all studied tissues, but they were more represented in the intestinal epithelium (Fig. 3H,I). Thus, the characteristic tissue specificity of certain bacteria was revealed, which is consistent with the results of the 16S rRNA analysis (Table S2). The most common bacteriocytes were formed by *Labrenzia* and *Stenotrophomonas*, while *Maritalea* and *Achromobacter* were detected less frequently.

The number of bacterial clusters detected by Eub388 was higher than that of clusters labeled with each of the group-specific probes. The total number of bacteriocytes detected by the group-specific oligonucleotide probes was generally comparable to the number of bacteriocytes detected by EUB338, which is consistent with the assessment of the phylogenetic composition of the symbionts’ population.

### TEM observations

**Figure 4:**
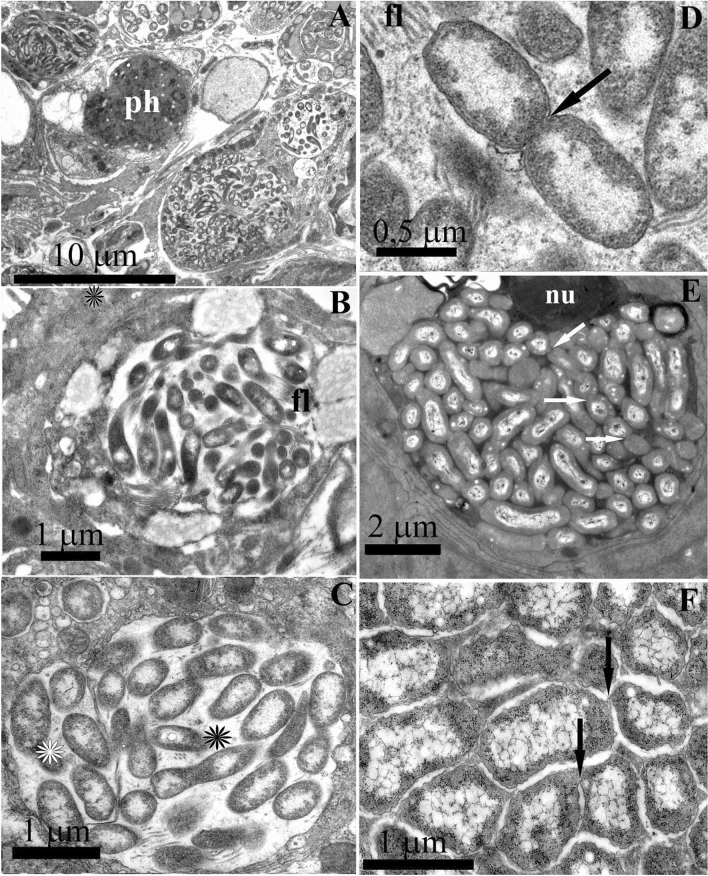
Endosymbiotic bacteria housed in bacteriocytes of *Rostanga alisae*. (**A**) Cells of integument epithelium of the foot containing bacteriocytes with several bacterial morphotypes of bacteria. Phagosome (ph) is visible. (**B**) Bacteriocyte containing curved-rod bacteria with lophotrichous flagella (fl). Vacuoles with electron-transparent contents are visible in the cytoplasm of bacteria. (**C**) Bacteriocytes containing rod-shaped bacteria with flagella (black asterisk) and without flagella (white asterisk). (**D**) Dividing flagellate bacterium in the bacteriocyte (arrow). (**E**) Bacteriocyte containing large rod-shaped bacteria, which forming a chain as a result of incomplete division. Arrows show the contacts of bacteria. Nucleus (nu) of the epithelial cell is visible. (**F**) Higher magnification of bacteria of this morphotypes indicated in (**E**). A tortuous outer membrane, extensive nucleoid zone, and vacuoles are visible. Arrows show the contact points of the outer membranes of adjacent individuals.

To examine the spatial organization of the bacterial symbionts, we performed TEM of the foot, notum, and intestine tissues. TEM revealed dense clusters of rod-shaped gram-negative bacteria enclosed in the epithelium covering the foot and notum of *R. alisae* (Fig. 4). Bacteria were separated by a membrane and located within specialized vacuoles, referred to as bacteriocytes, which could occupy most of cytoplasm of a host cell. The number of bacteria per bacteriocyte reached a few dozen (Fig. 4A–C). Two main bacterial morphotypes formed bacteriocytes; however, bacteria within each vacuole were monomorphic.

The most common bacteriocytes were those containing gram-negative curved-rod lophotrichous bacteria (0.3–0.4 μm) having up to six flagella (Fig. 4B,C). Less common were bacteriocytes with large gram-negative rod-shaped bacteria (0.7 × 1.5 μm), many of which appeared to be dividing (Fig. 4D–F). In some bacteriocytes, membranes of neighboring bacteria came into contact. As a result of incomplete division, the rod-shaped bacteria can form chains of individuals connected to each other, suggesting their clonal origin within a single specialized vacuole (Fig. 4E,F).

**Figure 5:**
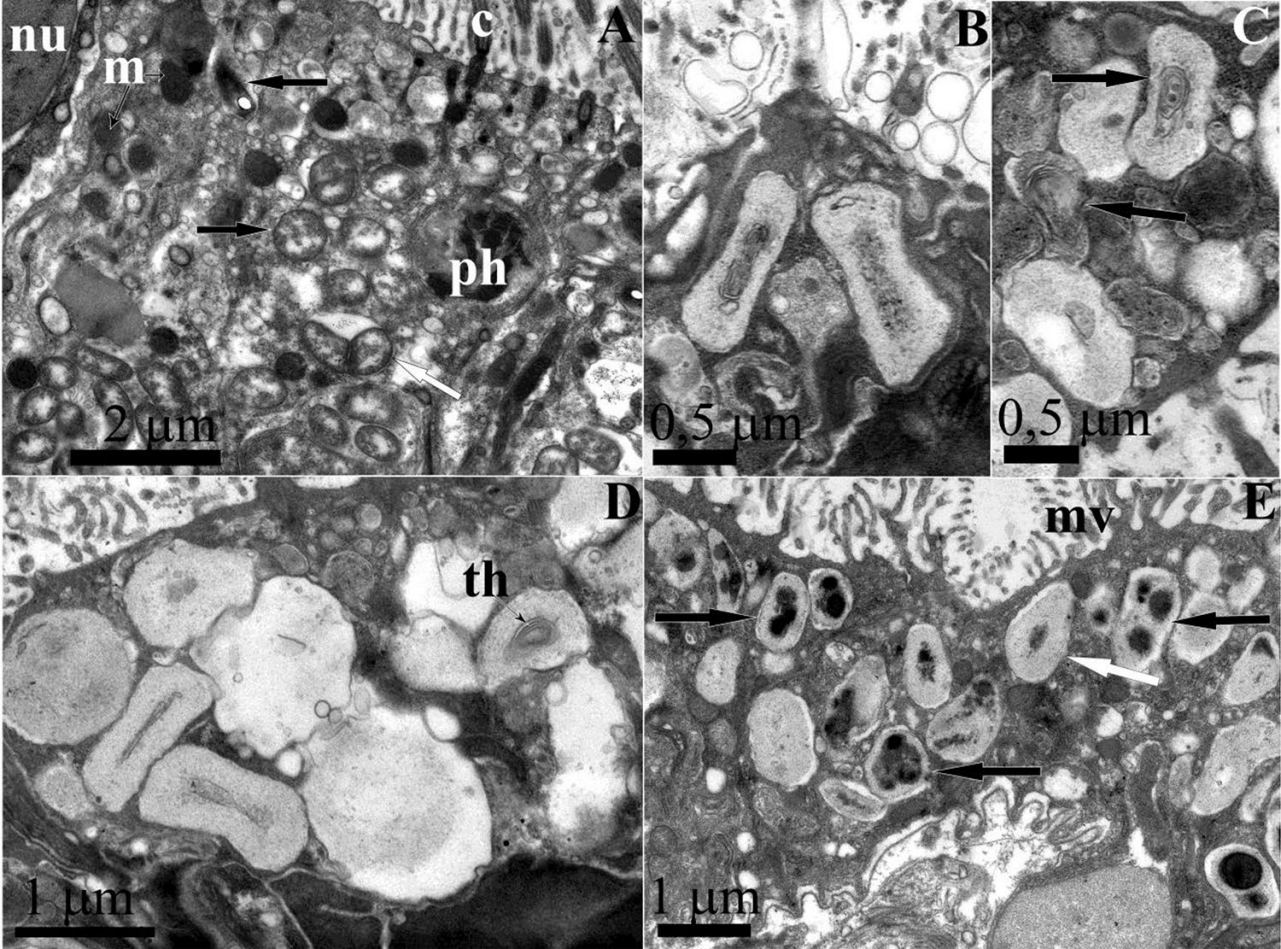
Endosymbiotic single bacteria of *Rostanga alisae*, TEM images. (**A**) Numerous Single gram-negative bacteria within secondary vacuole (light arrows) in the apical zone of the cytoplasm of the epithelium of the foot. As a result of division (dark arrow indicates a dividing bacterium), bacteriocytes contain two or more bacteria. Cilia (c), phagosome (ph), mitochondria (m), and nucleus (nu) of epithelial cells are visible. (**B,C**) Cyanobacteria (arrows) in the epithelium of the notum and (**D**) foot; a developed polysaccharide capsule and concentrically arranged membranes of the thylakoid type (th) are visible. (**E**) Different morphotypes of cyanobacteria containing inclusions in the intestinal epithelium cells: bacteria with a developed capsule (light arrow); bacteria with a narrow capsule (dark arrow). The apical surface of epithelial cells has microvilli (mv).

Epithelial cells also contained vacuoles with single gram-negative bacteria (Fig. 5A). Their morphotypes were diverse and similar to that described from bacteriocytes above. The similarity between the morphotypes of single bacteria and bacteria forming clusters, as well as the presence of dividing bacteria in the bacteriocytes, suggests that the observed single bacteria are the initial stage of formation of a dense bacterial cluster within bacteriocyte.

Apart from bacteriocytes packed with bacteria, large bacteria (diameter 0.5–0.7 μm, length up to 2 μm) with a well-developed polysaccharide envelope were found mainly in the intestinal epithelium and, more rarely, in the integumentary epithelium (Fig. 5B–E). Their cytoplasm contained concentrically arranged membranes of the thylakoid type, vacuoles, and granules (Fig. 5C–D). Bacteria were single or formed chains of several individuals (Fig. 5C,D). Some individuals in the colony had a thick, hypertrophied peptidoglycan fibrillar envelope (Fig. 5D). The presence of thylakoid membranes, the ability to form chains, and the hypertrophied peptidoglycan envelope in some individuals, which is typical of heterocysts, allows classifying these microbes as cyanobacteria.

**Figure 6:**
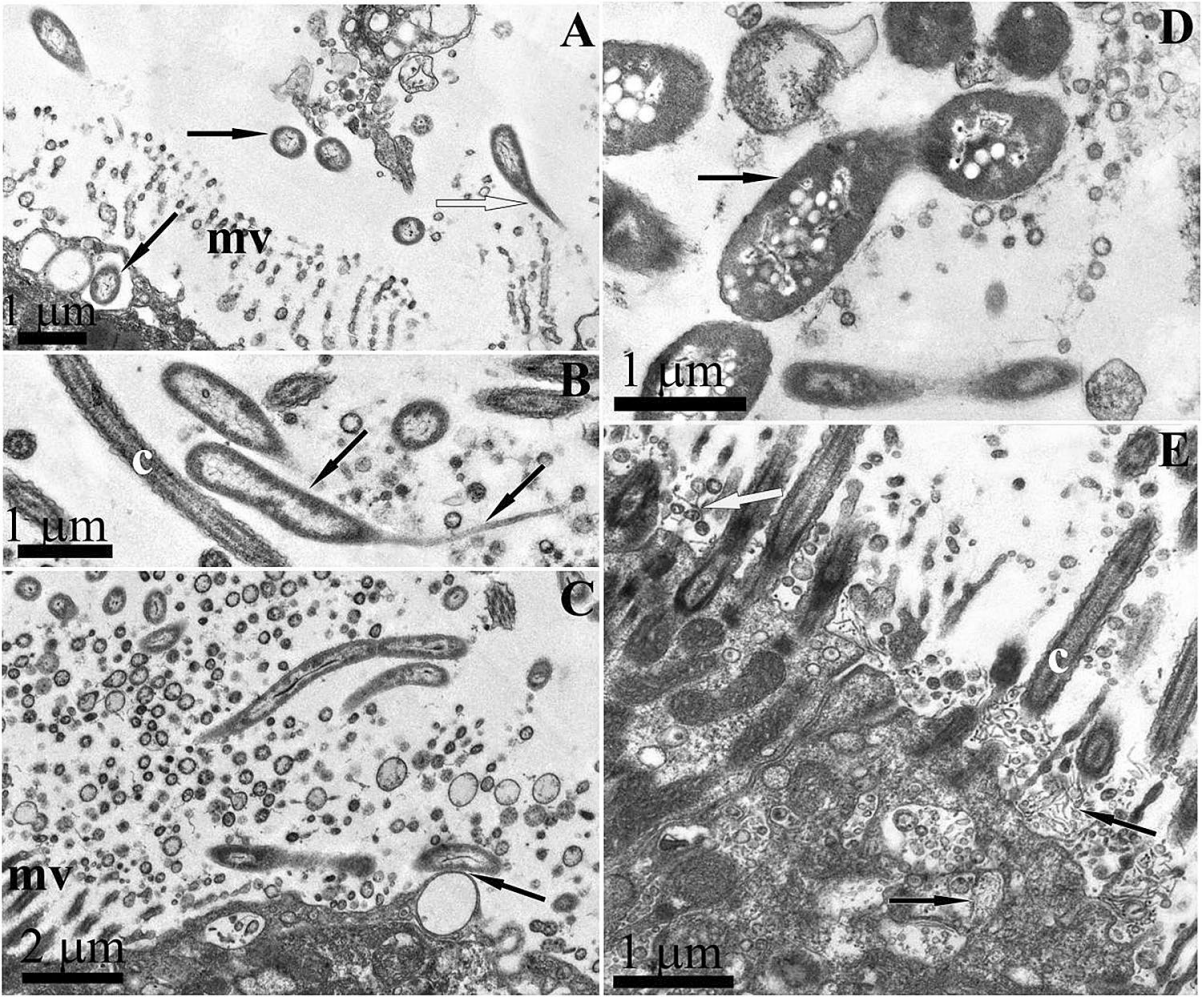
TEM images of epithelium cells and adjacent mucous layer of *Rostanga alisae* inhabited by exosymbiontic bacteria. (**A**) Bacteria having a long contact stalk-like extension directed towards the apical membrane of epithelial cells (white arrow). Dark arrows indicate similar bacteria, which are present both in the mucous layer and in vacuoles near the apical membrane of the integumentary epithelial cells. (**B**) The bacterium with a long process (dark arrows) in contact with the surface of the microvilli (cross section) of the integumentary epithelial cells. (**C**) Long rods localized between the microvilli (mv) of the integumentary epithelial cells. The arrow indicates the site of adhesion of the bacterial cell to the outer cell membrane. (**D**) Large bacteria with numerous electron-translucent vacuoles (dark arrow) (**E**) Processes of branching bacteria on the surface and in the cytoplasm of cilliated cells of the foot epidermis (dark arrows); branched processes are visible bearing spore-like vesicles on the surface (light arrow). Cilia (c) and microvilli (mv) are visible.

An ultrastructural analysis showed that the nudibranch bore various extracellular bacteria in the mucus layer of the foot and notum that were identified by a 16S rRNA gene sequence analysis as close relatives of the genera *Arthrobacter*, *Iamia*, *Ilumatobacter*, and *Kocuria* (Fig. 6). The most abundant were gram-negative curved rod-shaped bacteria (0.5 × 1.5–2 μm) (Fig. 6A) with a long (up to 1 μm) contact stalk-like extension, usually directed to the apical surface of epithelial cells and providing a primary contact of bacteria with the apical membrane of host’s epithelial cells before penetrating into cytoplasm (Fig. 6B). This confirms the presence of a similar bacterial morphotype in bacteriocytes in epithelial cells. Bacteria inhabiting the mucus layer often come into contact with the apical membrane of epithelial cells, which is probably the initial stage of their inoculation (Fig. 6C). Large gram-negative rods (0.7 × 3 μm) (Fig. 6D) with numerous electron-translucent vacuoles were much less common. Bacteria forming long branching filaments resembling fungal hyphae were observed on the surface of ciliated cells of the foot epithelium (Fig. 6E). Unusually thin, from 10 to 20 nm, non-septate filaments branched and formed round bubbles of 50–100 nm in diameter at the apex. Similar branching bacteria were also found in the apical cytoplasm of epithelial cells that indicates the presence of air and substrate mycelium of branching bacteria.

The presence of these symbionts was typical for all adult animals studied, although the pattern of distribution of bacteriocytes varied between individuals. The general occurrence of individuals with symbionts indicates an obligate rather than a facultative association between symbionts and the nudibranch.

### Fatty acid analysis

**Figure 7:**
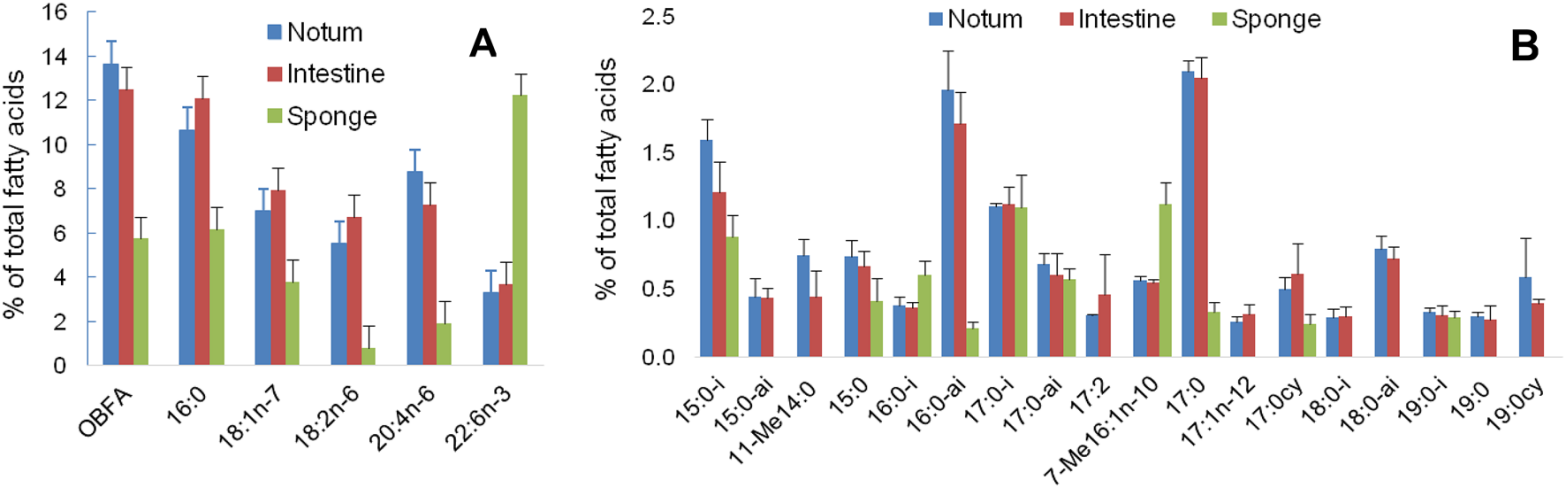
Distribution of the fatty acids among the notum and intestine of *Rostanga alisae* and its prey sponge *Ophlithaspongia pennata* (mean ± SD; n = 7). **(A)** Composition of principal fatty acids. **(B)** Composition of odd and branched fatty acids (OBFA) as markers of bacteria. Data on fatty acid composition and significant differences (one-way ANOVA) in their concentration between the nudibranch and its prey sponge are provided in Supplementary Table S3.

Fatty acid analysis is a useful tool to ascertain original nutrient sources acquired by partners of the association^5,32^. The integumentary tissues of notum and foot that were rich in symbiotic bacteria and the intestine with an abundance of cyanobacteria were analyzed separately but their fatty acid compositions showed no significant differences (Table S3). Both tissues exhibited exceptionally high concentrations of odd- and branched fatty acids (OBFA) typical for bacteria (13.7 and 12.5%, respectively), and also an abundance of saturated (19.6 and 20.8%) and monounsaturated fatty acids (20.8 and 21.4%), mainly due to 16:0 and 18:1n-7, along with high levels of n-6 polyunsaturated fatty acids (PUFA) (20.2–20.5%) (Fig. 7A). Marked differences were found between the nudibranch and its prey, the sponge *O. pennata*. The sponge contained the highest concentration of very long chain fatty acids (VLCFA) (28.1%). The nudibranch was much more enriched in PUFA n-6, in particular in linoleic acid (18:2n-6), 20:4n-6, and 22:4n-6, while PUFA n-3 such as 22:6n-3 and 22:5n-3, were more abundant in the sponge. The level of 18:2n-6, typical for cyanobacteria^33^, and essential for the mollusks, was almost by an order of magnitude higher in *R. alisae* than in the sponge. The difference in the OBFA content between nudibranch and sponge was also pronounced (Fig. 7B). Moreover, the composition of these bacterial markers differed between the nudibranch and its prey, the sponge. The most noticeable difference was in the content of 17:0 (2.1% in *R. alisae* vs. 0.3% in the sponge) and *anteiso*-16:0 (2.0% vs. 0.2%, respectively). The acids, *iso*-15:0, 17:1∆5, *iso*-18:0, *anteso*-18:0, and *cyclo*-19:0, were found only in *R. alisae*. The predominance of OBFA, 16:0, and 18:1n-7, which are typical for bacteria, as well as differences in the composition of bacterial markers between the nudibranch and the sponge *O. pennata* indicates the important bacterial contribution to the diet of the nudibranch.

It seems unlikely that the sponge *O. pennata* is the only source of fatty acids in *R. alisae*, since their levels in the predator nudibranch were significantly higher than in its prey sponge. *R. alisa*e tissues also contained twice as much bacterial marker *cis*-vaccenic acid (18:1n-7) as compared to that in the sponge. Moreover, some bacterial indicators (mainly *anteiso*-15:0, 11-Me 14:0, 7Me-16:1n-10, 17:0, 17:1n-12, *iso*-18:0, *anteiso*-18:0, 19:0, and *cyclo*-19:0) were identified only in the nudibranch, thus, confirming the different bacterial associations in the nudibranch and the sponge.

## Discussion

### Symbiont diversity and distribution

The present study provides the first evidence of symbiosis in *R. alisae*, a species of nudibranchs. This is the most multiple symbiosis that have ever been recorded for marine invertebrates. While many organisms establish an exclusively one-on-one relationship with a single microbial species or microbes belonging to the same functional group^5,12^, there are also organisms that harbor multiple microbial species, in which symbiont–symbiont and host–symbiont interactions occur. Six phylotypes of chemoautotrophic bacteria were reported for mussel *Idas* sp. from a cold seep area^11^ and five extracellular symbionts for the gutless oligochaete worm *Olavius algarvensis*^34^. However, in these cases, symbioses involving bacteria and marine invertebrates are either endosymbiotic microbes co-occurring inside the host bacteriocytes^5,11^ or ectosymbiotic microbes associated with the external surfaces of the animals^3,4,9,15,34^, with the exception of scaly-foot snail from hydrothermal vents having partnerships simultaneously with epi- and endosymbiontic microbes^35^.

Bacterial symbionts in *R. alisae* have appeared to be more diverse than was previously known for marine invertebrates. It is evident that the detected symbiont phylotypes differ greatly from all other known symbionts found in marine invertebrates. *Labrenzia* (Rodobacteriales) and *Maritalea* (Rhizobiales) have not been recorded as forming symbiotic associations with invertebrates or plants so far, although other members of the families Rodobacteriales and Rhizobiales are well known symbionts^14^. Strains of *Bradyrhizobium*, *Burkholderia*, *Achromobacter*, and *Stenotrophomonas* are reported as symbionts of plants, interacting with a vast majority of nodulating legume species and efficient in biological nitrogen fixation^36^. This may be important when considering the nature of these symbionts in the nudibranch. Symbioses between cyanobacteria and marine organisms are commonly found among marine plants, fungi, sponges, ascidians, corals, and protists^37,38^. *Synechococcus*, identified as dominant symbiont clones of *R. alisae* (Table S2), is a unicellular cyanobacterium common in the marine environment, providing a range of beneficial functions including photosynthesis, nitrogen fixation, UV protection, and production of defensive toxins^8,9,37^. Symbiotic interactions between actinobacteria and their host have been observed in insects, human, animals, and plants, where the bacteria provide the host with protection against pathogens and produce essential nutrients^39^. However, none of the members of the clade Actinobacteria recorded in *R. alisae* are known to live symbiotically.

### Arrangement of symbiotic association

Despite the high diversity of bacteria, they are well organized in the host. Dense clusters of rod-shaped bacteria, *Labrenzia*, *Maritalea*, *Bradyrhizobium*, *Burcholderia*, *Achromobacter*, and *Stenotrophomonas*, were found within host-derived vacuoles, referred to as bacteriocytes, inside epithelial cells of *R. alisae* (Fig. 3). Although such arrangement differs from that typical of bacteriocytes, which are usually considered as specialized cells of the hosts for harboring bacteria, it resembles that reported for scaly-food snail from hydrothermal vents, which harbor symbionts in the esophageal gland^35^. Bacteriocytes in the gastropod *Lurifax vitreus* found near hydrothermal vents also constitute a portion of the mantle epithelium; they have large vacuoles containing many live and dividing bacteria^40^. Each bacteriocyte was densely packed with certain symbionts, and the bacteriocytes were randomly distributed within the epithelium cells. A distinctly regular distribution pattern was observed in the gill epithelium of the mussel *Bathymodiolus* sp.: the thiotrophic symbionts occupy the apical region, and the methanotrophic symbionts are more abundant in the basal region of bacteriocytes^4^. In the mussel *Idas* sp., however, there is no spatial pattern of the six distinct bacterial phylotypes, and the symbionts are mixed within bacteriocytes^11^.

*Synechococcus* dominated the cytoplasm of intestinal epithelium and, more rarely, epidermis cells, mainly as specialized cell type referred to as nitrogen-fixing heterocysts. They are visually similar to cyanobacteria from corals and sponges^8,37^.

The phylogenetic diversity and the spatial organization of the symbiotic community in *R. alisae* were determined by the 16S rRNA analysis, which was consistent with the results of FISH and TEM. Unlike most symbioses of marine invertebrates when bacteria house specialized host cells^5,11^ or cover epidermis^7,15^, symbiotic association of *R. alisae* exhibited spatial partitioning between symbionts, which were unevenly distributed between the tissues (Table S2). It has been established that different members of the microbial community can complement each other in acquisition of various restrictive nutrients, confirming the importance of the functional diversity of symbionts^41^. Thus, *Stenotrophomonas rhizophila* and *Bradyrhizobium* build a beneficial association in the rhizosphere and can act synergistically on promoting growth and nutrient uptake of soybean^36^. Cyanobacteria can interact synergistically with beneficial members from the endophytic microbiome of rice seedlings^42^. The location of bacterium in the organism of *R. alisae* may, in fact, depend on the specific metabolic and ecological roles that the symbionts play, and also on the interaction with bacterium belonging to different physiological groups.

### Nature of symbiosis

Symbiotic associations between microbes and invertebrates are acquired mainly in a nutrient-depleted environment where symbionts usually provide their hosts with essential nutrients and high-energy compounds^1^. In contrast to known symbioses between microbes and gutless invertebrates, which obtain nutrients exclusively from the bacteria, *R. alisae*, like most nudibranch species, is a sponge-eating predator. However, due to the lack of adipose tissue, sponges are distinguished by a low lipid content (0.4 to 1.5% of wet weight)^43^ and also by specific proteinaceous spongin fibers and chitin, a polysaccharide similar to cellulose that can be indigestible for some predators, which together indicate their low nutritional value. Furthermore, *R. alisae* feeds exclusively on the sponge *O. pennata*; therefore, in habitats with low prey availability, this nudibranch has to survive starvation while searching for sponge assemblages. We suppose that symbiotic bacteria of *R. alisae* contribute to the utilization of low-quality food, similarly to symbiotic bacteria from the genera *Rhodobacter*, *Burkholderia*, and *Aeromonas* associated with the detritivorous isopod *Asellus aquaticus*^44^.

A fatty acid analysis, as a useful approach to clarifying the nature of symbiosis^5,20,32^, has confirmed the trophic interaction between symbionts and the nudibranch host (Table S2). Among the fatty acids of symbiotic bacteria in *R. alisae*, OBFA are a major acyl constituent of membranes in *Stenotrophomonas*^45^ and also in Actinobacteria, *Arthrobacter*, *Iamia*, *Ilumatobacter*, and *Kocuria*^46^. Cis-vaccenic acid is a major component of *Maritalea*^30^. Omega-cyclohexyl tridecanoic acid (cyclo19:0) is specific to *Bradyrhizobium*^47^, *Burkholderia*, and *Achromobacter*^48^. Linoleic acid is produced by cyanobacteria including marine species of *Synecoccocus*^33^; in nudibranch, it obviously serves as a precursor in the synthesis of arachidonic acid (20:4n-6), thus, providing additional evidence for the transfer of fatty acids from symbionts to the host. Mollusks are capable of converting linoleic acid to arachidonic acid, since they have enzymes required for its synthesis^21^. The presence of these bacteria-specific markers and the abundance of arachidonic acid confirm the metabolic role of symbionts in the nudibranch host.

Among nutrients, biologically available nitrogen can be considered a restrictive nutrient for the sponge-eating *R. alisae*, which can be acquired with the help of nitrogen-fixing symbionts, also referred to as diazotrophs. *R. alisae* harbors *Bradyrhizobium*, *Burkholderia*, *Achromobacter*, and *Stenotrophomonas* that are efficient in biological nitrogen fixation previously found to be associated with nodulating legume species^36^. Symbiotic nitrogen fixers are known to be associated with a variety of marine invertebrates such as wood-boring bivalves, corals, sponges, sea urchins, tunicates, and polychaetes^7,8,37^. Moreover, the protection of the enzyme nitrogenase that catalyzes N_2_ fixation against oxygen is an important physiological requirement in bacteria such as symbiotic *Bradyrhizobium*, *Burkholderia*, *Achromobacter*, and *Stenotrophomonas* that are located in bacteriocytes and provide this protection. *Synechococcus* is known as a nitrogen-fixer^37,49^. It performs N_2_ fixation in heterocysts where nitrogenase is restricted under oxic conditions. Indeed, heterocysts of *Synechococcus* are abundant in the intestine cells of *R. alisae* (Fig. 5B–D).

Nitrate assimilation is one of the major processes of nitrogen acquisition by many heterotrophic bacteria and cyanobacteria^50,51^. Symbionts of *R. alisae* can play an important role in the process of nitrate utilization through denitrification, dissimilatory nitrate reduction, and assimilatory nitrate reduction as a nitrogen source and synthesize it into organic nitrogen. The nitrate reducers, *Labrenzia*^52^, *Stenotrophomonas*^53^, *Maritalea*^30^, and Rhodobacteraceae^29^ are widely represented in *R. alisae*. *Synechococcus* also utilizes nitrate, nitrite, or ammonium for growth^50^. Thus, symbiotic bacteria may play a significant role in the N-budget of the nudibranch mollusk.

The symbiotic bacteria of *R. alisae*, including *Bradyrhizobium*, *Maritalea*, *Labrenzia*, *Burkholderia*, *Achromobacter*, *Stenotrophomonas*, *Arthrobacter*, *Iamia*, *Ilumatobacter*, and *Kocuria*, are known as carboxydotrophic or carbon monoxide (CO) oxidizers^54,55^. Despite the toxicity of CO for multicellular organisms, numerous aerobic and anaerobic microorganisms can use CO as a source of energy and/or carbon for cell growth^56^. The marine worm *Olavius algarvensis* establishes symbiosis with chemosynthetic bacteria using CO, a substrate previously not known to play a role in symbiotic associations with marine invertebrates, as an energy source^57^. We do not rule out that the *R. alisae* symbionts also might exploit CO as carbon and energy source. Despite this, assumption may seem impossible taking in account the CO toxicity, but, since many invertebrates (mollusks, tube worm, etc.) use toxic sulfate, thiosulfate, and methane as an energy source^1,15^, this hypothesis is worth to be addressed.

An important component of skeleton in marine sponges of the family Microcionidae, including *O. pennata*, is the structural polysaccharide chitin^58^. Some bacteria are capable of hydrolyzing chitin via the activity of chitinolytic enzymes and can utilize chitin as a source of carbon, nitrogen, and/or energy^59^. Chitinase activity was documented for strains of *Labrenzia*^60^, *Burkholderia*^61^, *Arthrobacter*^62^, *Achromobacter*^63^, *Stenotrophomonas*^64^, *Alcaligenes*^65^, and actinobacteria^59^ associated with *R. alisae*. Thus, these bacteria can work synergistically to digest chitin and spongin, contributing to feeding success of the host nudibranch which depends solely on low-quality, nitrogen- and carbon-deficient food available.

Furthermore, direct evidence has confirmed that many bioactive compounds from invertebrates are produced by symbiotic microorganisms^66,67^. Many biologically active compounds including toxic and deterrent secretions have been identified in nudibranchs of the family Discodorididae^68^. Symbiotic bacteria may exhibit toxic activity to provide the host nudibranch with chemical defense against predators and environment. Bacteria, especially actinobacteria, living in a symbiotic relationship with *R. alisae* may help the host in defense, since nudibranch lack a shell, and secondary metabolites of bacteria can provide them with chemical defense against predators and environment, as has been reported for some marine invertebrates^2,9,10^.

In complex associations, the integration and coexistence of symbionts depend on supplementary partnerships and mutual contribution to the host’s metabolism^41^. The most intensively studied cases are highly specialized associations, where both partners can only exist in close relationship with one another. The relatively high diversity of microbes in *R. alisae* complicates understanding the complex pattern of molecular and cellular interactions between the host and its symbionts.

## Conclusion

The current study demonstrates that the nudibranch *R. alisae* is a unique species among the marine animals, as it harbors a wide range of phylogenetically distinct but physiologically similar bacteria. These bacteria, known as aerobic chemoheterotrophs or facultative anaerobes, are capable of nitrogen fixation and/or denitrification under anaerobic conditions, oxidizing of carbon monoxide, hydrolyzing of chitin, and can use chitin as a source of carbon, nitrogen, and/or energy. They may also exhibit toxic activity and may provide chemical defense for the host nudibranch. A combination of such characteristic features as the dense population of diverse endo- and extracellular bacteria, the specific diet that is extremely poor in organic nutrients and energy, and the abundance of diverse fatty acids specific for bacteria and cyanobacteria in its composition suggest the existence of nutritional symbiosis in this nudibranch species. Overall, the data obtained expand our understanding of the biological role of the symbiosis of Metazoa with bacteria.

## Supplementary Information

Supplementary Tables.

## Data Availability

The datasets generated or analyzed during the current study are included in this published article and its Supplementary Information files. All sequences are available in GenBank under accession numbers MZ410589–MZ410616.
