# Supplementary material for: Multiple bacterial partners in symbiosis with the nudibranch mollusk Rostanga alisae
Source: Sci Rep. 2022 Jan 7;12:169. doi: 10.1038/s41598-021-03973-7 (PMC8742107; doi:10.1038/s41598-021-03973-7)
Supplement: Supplementary file 1 — Supplementary Tables. [file 41598_2021_3973_MOESM1_ESM.pdf]

Multiple bacterial partners in symbiosis with the nudibranch mollusk *Rostanga alisae*

Natalia V. Zhukova, Marina G. Eliseikina, Evgeniy S. Balakirev & Francisco J. Ayala

Table S1. Oligonucleotide probes for Fluorescence *in situ* hybridization analysis.

Probe (genus)	Nucleotide sequence (5'→3')
Labrenzia (#1095)	5’-ACATTTGGTGCTACATTCGGAGACGGATGGTTCCCTTCGGGGACGCCAGG-3’
Maritalea (#1095)	5’-ACATACCTATCGCAGTCTCCAGAGATGGAGACCTTCAGTTCGGCTGGATAGGATA-3’
Iamia (#887)	5’-GTAAACGTTGGGCACTAGGTGTGGGGTCTATTCAACGGA-3’
Ilumatobacter (#887)	5’-GTAAACGTTGGGCACTAGGTGTGGGTCTCAACCAACGAGA-3’
Burkholderia (#487)	5’-TCCGGAAAGAAATCATCCTGGCTAATATCCGGGGTGGA-3’
Bradyrhizobium (#1093)	5’-TCCCGGTCGCGGACTCCAGAGACGGAGTTCTTCAGTTCGGCTGGACCGG-3’
Arthrobacter (#1087)	5’-AAGGCTTGACATGAACCGGAAACACCTGGAGACAGGTGCCCCGCTTGCGGTCGGTTT-3’
Achromobacter (#648)	5’-TCGGAAAGAAAGATGTGAAATCCCAGAGCTTAACTTTGGAACTGCATTTTTAACTAC-3’
Stenotrophomonas (#449)	5’-TGGGTGAAGAAGGCCTTCGGGCTGTAAAGCCCTTTTGTTGGGAAAGAAATCCAG-3’
Cyanobacteria (#872)	5’-TGTAGTCCTGGCCGTAAACGATGAACACTAGGTGCCGGGGGAATCGACCCCTCC-3’
A bacterial universal probe set EUB338	5’-GCTGCCTCCCGTAGGAGT-3

Table S2. Diversity of bacteria associated with the nudibranch *Rostanga alisae* tissues. Total number of phylotypes (using 97% criterion of similarity) for each tissue is shown in parentheses. Values in bold represent the most dominant bacterial group found in association with source.

	Foot	Intestine	Notum	Total
**α-Proteobacteria**				**15**
Bradyrhizobiaceae	+			1
Rhodobacteraceae	+ + +			3
Bradyrhizobium		+	+ + +	4
Maritalea	+ + +			3
Labrenzia	+ + + +			4
**β-Proteobacteria**				**13**
Alcaligenaceae	+		+	2
Burkholderia		+	+ + +	4
Achromobacter	+ +	+ +	+ +	6
Wautersia	+			1
**γ-Proteobacteria**				**16**
Acinetobacter			+	1
Aliivibrio		+		1
Legionella		+ +		2
Coxiella		+		1
Lysobacter	+		+	2
Aquicella	+	+		2
Stenotrophomonas	+ + + +		+ + +	7
**δ-Proteobacteria**				
Desulfovibrio	+			1
**Cyanobacteria**				**19**
Synechococcus	+ + + + + +	+ + + + + + + + + + +	+ +	19
**Actinobacteria**				**7**
Arthrobacter			+ + +	3
Iamia	+			1
Ilumatobacter	+ +			2
Kocuria	+			1
**Planctomycetes**				
Planctomycetes		+		1
**Fusobacteria**				
Leptotrichia			+ +	2

Table S3. Fatty acid composition (% of the total fatty acids) of the nudibranch tissues *Rostanga alisae* and its prey sponge *Ophlithaspongia pennata* (mean values ± standard deviation; n=7). Sing * indicates insignificant difference between the values.
The fatty acids that contributed to less than 1.0% of the total fatty acids were omitted from the table. Among them: Odd and branched fatty acids: 14:1Δ5, anteiso-15:0, branched-15:0, 15:0, iso-16:0, anteiso-17:0, 17:2, 7-Me-16:1n-10, 17:1Δ5, cyclo-17:0, iso-18:0, anteiso-18:0, iso-19:0, 19:0, cyclo-19:0.
Very long chain fatty acids: 24:0, 24:1Δ9, 25:0, 25:1Δ19, 25:3Δ5,9, 26:2Δ5,19, 27:2Δ5,9.

**Fatty acids**	***Rostanga alisae***		***Ophlithaspongia pennata***
	**Notum**	**Intestine**	
14:0	1.7 ± 0.2	1.6 ± 0.2	0.7 ± 0.1
15:0-*iso*	1.6 ± 0.2	1.2 ± 0.2	0.9 ± 0.2
16:0-*anteiso*	2.0 ± 0.3	1.7 ± 0.2	0.2 ± 0.0
16:0	10.7 ± 0.8	12.1 ± 1.5	6.2 ± 0.2
16:1n-7*	1.3 ± 0.2	1.3 ± 0.3	1.1 ± 0.1
17:0-*iso**	1.1 ± 0.0	1.1 ± 0.1	1.1 ± 0.2
17:0	2.1 ± 0.1	2.0 ± 0.2	0.3 ± 0.1
18:0	6.4 ± 0.3	6.2 ± 0.5	2.3 ± 0.1
18:1n-11	1.6 ± 0.2	1.2 ± 0.3	0.3 ± 0.0
18:1n-9	5.2 ± 1.0	5.3 ± 0.8	1.7 ± 0.1
18:1n-7	7.0 ± 0.4	8.0 ± 1.3	3.8 ± 0.0
18:2n-6	5.5 ± 0.9	6.7 ± 0.7	0.7 ± 0.1
20:1n-7	1.6 ± 0.3	1.7 ± 0.5	–
20:2Δ5,11	1.2 ± 0.2	1.0 ± 0.4	0.3 ± 0.1
20:2Δ5,13	8.4 ± 1.4	6.1 ± 1.6	0.3 ± 0.1
20:4n-6	8.8 ± 0.7	7.3 ± 1.0	1.9 ± 0.3
20:5n-3*	1.9 ± 0.2	1.9 ± 0.2	1.9 ± 0.4
22:1n-7	0.5 ± 0.1	0.4 ± 0.2	2.4 ± 0.0
22:4n-6	4.0 ± 0.4	4.6 ± 1.3	3.9 ± 0.2
22:5n-3	2.6 ± 0.5	3.0 ± 0.7	6.2 ± 0.4
22:6n-3	3.3 ± 0.6	3.7 ± 0.7	12.2 ± 0.7
24:2Δ5,9	0.4 ± 0.1	0.4 ± 0.1	2.2 ± 0.8
25:2n-5,9	0.4 ± 0.3	0.5 ± 0.1	3.0 ± 0.2
26:2Δ5,9	4.5 ± 0.5	5.2 ± 0.5	28.2 ± 0.6
26:3Δ5,9,19	0.7 ± 0.1	0.8 ± 0.2	5.2 ± 0.8
27:2Δ5,9,15	0.7 ± 0.3	1.0 ± 0.4	4.2 ± 0.4
